# Cyberchondria, Health Literacy, and Perception of Risk in Croatian Patients with Risk of Sexually Transmitted Infections and HIV—A Cross-Sectional Study

**DOI:** 10.3390/epidemiologia5030036

**Published:** 2024-08-22

**Authors:** Tanja Staraj Bajcic, Iva Sorta-Bilajac Turina, Marko Lucijanic, Tamara Sinozic, Mirela Vuckovic, Ksenija Bazdaric

**Affiliations:** 1 Department of Epidemiology of Communicable and Chronic Non-Communicable Diseases, Teaching Institute of Public Health of the Primorje—Gorski Kotar County, Kresimirova 52 a, 51000 Rijeka, Croatia; tanja.staraj-bajcic@zzjzpgz.hr; 2 Department of Public Health, Teaching Institute of Public Health of the Primorje—Gorski Kotar County, Kresimirova 52 a, 51000 Rijeka, Croatia; iva.sorta-bilajac@zzjzpgz.hr; 3 Department of Environmental Medicine, Faculty of Medicine, University of Rijeka, Brace Branchetta 20, 51000 Rijeka, Croatia; 4 Division of Haematology, University Hospital Dubrava, Av. Gojka Suska 6, 10000 Zagreb, Croatia; 5 Internal Medicine Department, School of Medicine, University of Zagreb, Salata 3, 10000 Zagreb, Croatia; 6 Private Family Medicine Practice, Barba Rike 5a, 51417 Moscenicka Draga, Croatia; tamara.sinozic@medri.uniri.hr; 7 Department of Family Medicine, Faculty of Medicine, University of Rijeka, Brace Branchetta 20, 51000 Rijeka, Croatia; 8 Department of Physiotherapy, Faculty of Health Studies, University of Rijeka, Viktora Cara Emina 5, 51000 Rijeka, Croatia; mirela.vuckovic@fzsri.uniri.hr; 9 Department of Basic Medical Sciences, Faculty of Health Studies, University of Rijeka, Viktora Cara Emina 5, 51000 Rijeka, Croatia

**Keywords:** cyberchondria, health literacy, risk assessment, health risk behaviours, sexually transmitted infections

## Abstract

**Background:** The perception of the risk of sexually transmitted infections (STIs) is a well-researched phenomenon, but not in relation to cyberchondria and health literacy. Therefore, our aim was to examine the association between the assessment of the risk of sexually transmitted diseases and HIV, cyberchondria, and health literacy. **Materials and methods**: This study was conducted in 2020–2021 in Croatia. The experimental group consisted of patients from the Centre for Voluntary Counselling and Testing for HIV (N = 134). The control group consisted of other patients recruited from daily epidemiological practices (N = 171). The instruments that were used were questionnaires that measure the perception of risk of sexually transmitted infections and HIV, health literacy, and cyberchondria. **Results**: Out of 305 respondents, 134 (43.9%) were in the STI counselling group, and 171 (56.1%) were in the control group. Generally, the participants in the former perceived the risk of sexually transmitted infections and HIV to be higher (but still low) than those in the latter. There was no difference in health literacy between the STI and control group; the health literacy score was average in both groups. There were differences in almost all the cyberchondria factors, except for Mistrust. Health literacy, belonging to the STI counselling group, and risk behaviours were positively associated with CH in the logistic regression analysis. **Conclusions**: People with a risk for STIs tend to minimize this risk, have higher cyberchondria levels, and have average health literacy. Cyberchondria is positively associated with health literacy, and more education about excessive health-related internet research is needed.

## 1. Introduction

### 1.1. Sexually Transmitted Infections as a Public Health Challenge

Every day, more than a million sexually transmitted infections (STIs) are acquired worldwide. It is estimated that, in 2020, there were 374 million new infections, with 1 in 4 being STIs: chlamydia (129 million), gonorrhea (82 million), syphilis (7.1 million), and trichomonas (156 million). In 2016, over 490 million people were estimated to be living with genital herpes (HSV-2). In 2019, human papillomavirus (HPV) caused over 620,000 new cancer cases in women and 70,000 in men. Viral hepatitis is considered a major public health challenge of this decade, with 254 million people living with hepatitis B and 50 million living with hepatitis C in 2022 [1]. It is estimated that, by the end of 2022, there were 1 million people infected with HIV worldwide, 1.3 million newly infected, and 630,000 people who died as a result of the terminal stage of HIV infection—AIDS [2].

In Croatia, HIV infection has been monitored since 1985, when the first patient infected through a blood transfusion was registered. The HIV registry at the Croatian Institute of Public Health (HZJZ) has so far recorded 2017 infected people, of whom there are 613 (30%) AIDS cases and 256 (13%) deaths [3]. According to the Registry of Infectious Diseases, in the last 5 years, there were 129 chlamydia, 38 hepatitis B, 98 hepatitis C, 37 syphilis, and 24 gonorrhea estimated cases per year [4].

### 1.2. Centres for Voluntary, Anonymous, and Free HIV Counselling and Testing

The Republic of Croatia is a country with a low risk of an HIV epidemic, partly thanks to the establishment of 10 centres for voluntary, anonymous, and free HIV counselling and testing (VCT) during the operation of the Global Fund to Fight Tuberculosis, AIDS, and Malaria (GFATM) since 2003–2006 [5]. A VCT was established in Rijeka in 2004 and represents an important segment of the work of the Department of Epidemiology of Communicable and Chronic Non-Communicable Diseases of the Teaching Institute of Public Health of Primorje–Gorski Kotar County (NZZJZ PGŽ). The possibility of voluntary, anonymous, and free counselling on the presence of antigens/antibodies (HIV) and hepatitis B and C viruses in the patient’s blood is an important component in preventing the spread of sexually transmitted infections (STIs) and an entry point towards treatment for every newly diagnosed patient detected using VCT [6]. Annually, about 300 people are tested using VCT, of which a third are men who have sex with men (MSM). The results of randomized clinical studies indicate that the possibility of infection transmission is reduced in the MSM population if pre-exposure prophylaxis with antiretroviral drugs (PrEP) is used [7,8]. Considering the availability of PrEP, there is a possibility of excessive relaxation, that is, a low perception of the risk of the disease and the spread of HIV [9]. Gallagher et al., (2014), on a sample of 629 MSM in 505 (80.3%) cases, set an indication for taking HIV PrEP, although 366 (78%) respondents did not perceive their risk as significant enough to use it [9].

### 1.3. Health Literacy, Cyberchondria, and Risk Perception of Sexually Transmitted Infections

Health literacy (HL) is a concept defined as “the cognitive and social skills which determine the motivation and ability of individuals to gain access to, understand and use information in ways which promote and maintain good health” [10]. For example, Thompson et al. showed that a higher level of HL is positively associated with desirable health behaviour and, thus, those who knew that HPV was a health risk performed regular Pap tests [11]. However, a large European study of eight countries (n = 8000) showed that HL levels were insufficient [12].

Today, many patients use online resources as a source of information in cases of health problems. Although positive examples of using the internet for self-diagnosis have been recorded, in some cases it can lead to excessive, often unnecessary concern for one’s health when a medical professional is not consulted [13]. Fear of illness may be associated with excessive internet browsing, which interferes with daily functioning and is a predisposing factor for cyberchondria (CH) [13]. CH is an anxiety disorder characterized by excessive research of health content on the Internet [14]. The term was used for the first time in an article in the *Business Wire* newspaper in the United Kingdom in 1996, while in scientific communication, it was first used in an article published in *The Medical Journal of Australia* in 2000 [15]. It is a relatively new construct in the literature, so a search of the Medline database in PubMed using the keyword “cyberchondria” yields merely 171 records (searched on 17 June 2024).

Searching the internet for health information can result in a decrease or increase in anxiety. If the research is excessive and repetitive, it represents CH [16]. The symptoms of CH vary from the need for constant checking and looking for evidence and arguments to a heavy tolerance of uncertainty and a forced (compulsive) need for reassurance [13,16]. There is a risk of developing psychosomatic disorders and unnecessary costs in healthcare and it represents an issue in public health [13]. Compulsive thoughts disrupt the flow of regular thinking and occupy a dominant position. Therefore, the intensity of attention also changes, resulting in hypertenacity (permanently focused attention), hypovigilance (impaired ability to pay attention to something new), and distraction. The ability to think concretely may be reduced [13]. Poor perception and a negative interpretation of somatic sensations can lead to the stimulation of further mental and somatic psychopathological processes [13,14,15,16].

McElroy and Shevlin (2014) were the creators of the first Cyberchondria Severity Scale (CSS). They were motivated by the calculation of the economic burden and the loss of around GBP 3 million due to unnecessary visits to health institutions as a result of self-hyperdiagnosis, so they created the questionnaire [17]. The Croatian version was translated and validated by Begić et al. [18].

Tyrer et al. registered an increased prevalence of CH among patients in clinics in Great Britain during 2019, which caused an increase in the number of unnecessary health consultations. Education is very important in the prevention of CH, either individually or through public health services and the media [19].

The perception of the risk of STIs is a well-researched phenomenon, but not in relation to CH and HL. According to our knowledge, the relationship between CH, HL, and the perception of STI and HIV risk is not available in the literature. Therefore, the aim of this study was to investigate the relationships between cyberchondria, the perception of the risk of sexually transmitted infections, and health literacy. We expected that participants with a higher risk of STIs would have a lower HL and a higher CH.

## 2. Materials and Methods

### 2.1. Study Design and Period

This non-randomized controlled trial with a cross-sectional study design was conducted at the Department of Epidemiology of Communicable and Chronic Non-Communicable Diseases of the Teaching Institute of Public Health of Primorje–Gorski Kotar County, Croatia, from January 2020 to December 2021.

### 2.2. Procedure

Participants were divided into two groups (n = 305 in total) and anonymously and voluntarily filled out questionnaires on the risk perception of STIs and HIV, the eHealth Literacy Scale, and the Cyberchondria Severity Scale during the pre-test counselling process (STI counselling group, n = 134) and during the examination (control group, n = 171).

#### 2.2.1. The STI Counselling Group Procedure

The VCT process consisted of anonymous and voluntary pre-test counselling, STI and HIV testing, and post-test counselling. When entering the VCT procedure, the patient received their unique code, regardless of whether they were participating in this research study.

Pre-test counselling included a conversation with the patient and a doctor’s risk assessment and indication for STIs and HIV laboratory tests. The results of the laboratory tests were available within a week upon presentation of a uniquely coded referral to the counsellor during VCT working hours. Collecting the findings was voluntary and the responsibility of the patient. The resulting code remained the same throughout the procedure.

In the pre-test counselling process, patients were asked to participate in this research regardless of the doctor’s assessment or the need for testing. Patients/respondents who agreed to participate independently filled out three questionnaires under the same code. To further ensure data confidentiality, the initial survey was copied and attached as part of the research documentation. The original initial survey was archived.

#### 2.2.2. The Control Group Procedure

Patients from the control group were asked to fill out three coded questionnaires after the examination.

### 2.3. Materials

#### 2.3.1. Laboratory Tests

Patients had their blood drawn at the Department of Clinical Microbiology of the Teaching Institute of Public Health of Primorje–Gorski Kotar County, where the blood samples were analyzed. Samples were tested for the presence of antigens/antibodies to HIV, HBV, and HCV.

The previously created unique code was also used when analyzing the samples in the Laboratory for Serodiagnostics of the Department of Clinical Microbiology.

#### 2.3.2. Questionnaires

Three questionnaires were used in this research: the Counsellor’s assessment of risk behaviours of respondents, the eHealth Literacy Scale, and the Cyberchondria Severity Scale.

1. Counsellor’s assessment of risk behaviour

The Counsellor’s assessment of risk behaviour questionnaire was used in the work of VCT in the standard procedure [6]. It consists of mandatory and optional parts. The first contains the following: demographic data, basic data on circumstances of an increased risk for STI, the decision to test, the test result, and post-test counselling (referral to medical, social, or psychological help). The second contains 23 questions related to risks: source of information, marital status, sexual orientation, use of contraception and other risks, and doctor’s risk assessment.

2. The eHealth Literacy Scale

The eHealth Literacy Scale (eHeals) by Norman and Skinner from 2006 is a self-assessment measure that includes an individual’s ability to read, use a computer, search for information, understand health information, and put that information into context [20]. The translation into Croatian was made by Prof. Jokić Begić with colleagues, who gave her consent to use the translation of the scale via e-mail.

The score was defined as the total sum of the responses. The range of results was from a minimum of 8 to a maximum of 40 points. HL was considered low if the score was 8–18 points, average if it was 19–29 points, and high if it was 30–40 points. Cronbach’s alpha as a measure of this scale’s reliability was α = 0.94.

3. Cyberchondria Severity Scale (CSS)

The Cyberchondria Severity Scale (CSS) by McElroy and Shevlin is intended to measure anxiety resulting from excessive internet research of health symptoms, i.e., cyberchondria. After validation, a questionnaire was designed for patients with good psychometric properties of validity and high reliability, with a Cronbach’s α of 0.80 [17]. With 33 items, it measures the following: (1) Compulsion (8 items): disruptive, unpleasant, and compulsive search characteristics; (2) Distress (8 items): negative emotional states when searching; (3) Excessiveness (8 items): unnecessary and repeated searches for the same symptoms and diseases; (4) Reassurance (6 items): need for reassurance provided by a qualified person; and (5) Mistrust of Medical Professional (3 items): a person’s internal conflict about whether to trust health professionals or themselves [17].

The total score can range from 33 to 165 points. CH was considered low if the score was 33–76, average if it was 77–121 points, and high if it was 122–165 points.

For each scale, the total score was divided by the number of items, so the range of results was 1–5 and comparable. Low results were considered if the total score was 1–2.33, average if it was 2.34–3.67, and high if it was 3.68–5.00.

Prof. Jokić Begić and colleagues translated the questionnaire [18] and gave consent for the use of the translation of the CSS via e-mail. Cronbach’s alpha for the total scale was α = 0.91, with the subscale reliabilities ranging from α = 0.68 (Mistrust of Medical Professionals) to 0.91 (Compulsion; Distress)

### 2.4. Participants

The sample consisted of a total of 305 participants divided into STI counselling and control groups.

#### 2.4.1. STI Counselling Group

This group (n = 134) consisted of patients who visited the Department of Epidemiology of Communicable and Chronic Non-Communicable Diseases of the Teaching Institute of Public Health of Primorje–Gorski Kotar County, Croatia, for voluntary, anonymous, and free HIV counselling and testing (VCT).

##### Inclusion Criteria

All adult patients of both sexes who visited the VCT centre and obtained written informed consent were included in this study.

##### Exclusion Criteria

All minors of both sexes, patients who had a guardian and were incapable of independently filling out the questionnaire, and illiterate patients were excluded from this study.

#### 2.4.2. The Control Group

The control group (N = 171) consisted of patients from the department recruited from everyday epidemiologic practices: vaccinations (according to indications or commercial), passenger counselling related to vaccinations and measures to protect against infectious diseases during travel, health examinations of persons subject to mandatory health surveillance, anti-rabies treatment of persons (bitten/injured by animals), health surveillance of returnees from countries under increased risk of certain infectious diseases according to the current epidemiological situation or by order of border sanitary inspection, and a number of other activities that depend on the current epidemiological situation.

##### Inclusion Criteria

All adult patients recruited from everyday epidemiologic practices of both sexes who gave written informed consent were included.

##### Exclusion Criteria

All VCT patients, STI patients, minors of both sexes, persons who had a guardian and were unable to independently fill out the questionnaire, and illiterate persons were excluded from this study.

### 2.5. Statistical Analysis

#### 2.5.1. Sample Size Calculation

Power analysis for estimation of the required sample size was carried out prior to this study, with the assumptions of an error type 1 of 0.05, 90% power, a clinically significant difference in means of 10 points on the Cyberchondria Severity Scale between groups, and a standard deviation of 20 points in each group. The required sample size for obtaining a statistically significant difference under these assumptions was calculated to be at least 86 patients per group. This study enrolled a larger number of patients to account for potentially missing data and provide sufficient power for additional analyses.

#### 2.5.2. Analysis

Categorical data are presented as the frequency and relative frequency and were compared using the χ^2^ test. Quantitative data are presented as the mean and standard deviation and were tested using the *t*-test for independent samples for two groups. The average values of variables that had a non-normal distribution (tested with the Kolmogorov–Smirnov test) are presented with the median and interquartile range and were compared using the Mann–Whitney U test. The multivariate analysis of predictors of CH and its domains was carried out using logistic regression analysis, where CH and its domains were stratified at the median value. The Enter method was used for all regression analyses, synchronously evaluating all included independent variables.

Data were collected and stored in a database in MS Excel. Statistical data processing was performed using MedCalc ver. 16.2.1. (MedCalc Software, Ostend, Belgium). The level of statistical significance was set at 5% (*p* < 0.05).

### 2.6. Ethical Aspects

Before the start of this research, the epidemiologist/investigator obtained written informed consent from all participants. In the “Participants’ Information” document, they could read all the relevant information regarding the research aim, scope, design, and implementation, which emphasized their voluntariness, and the process of data collection, management, and protection according to contemporary bioethical guidelines. The investigator was always available for any clarification. This research received ethical approval from the committees of both the Teaching Institute of Public Health of Primorje–Gorski Kotar County, Croatia, and the Faculty of Medicine, University of Rijeka, Croatia.

## 3. Results

### 3.1. Participants’ Characteristics

There were 305 respondents in total, 134 (43.9%) in the STI counselling group and 171 (56.07%) in the control group (Table 1). The response rate from the invited participants was 100%.

The participants’ average age was 32 ± 10 in the STI counselling group and 36 ± 11 in the control group (*p* = 0.002). The gender distribution in the control group was quite even: 86 (50.3%) participants were female and 85 (49.7%) were male. However, in the STI counselling group, there were more male (n = 88, 65.7%) than female (n = 42, 31.3%) participants. Therefore, the test for gender distribution in the two groups was statistically significant (*χ*^2^ = 9.77; *p* = 0.002) and indicated that the gender distribution was not random in the control and STI counselling groups.

Most participants had at least a high school education (n = 105, 61.4% for the control; n = 87, 64.9% for the STI counselling group), and many had finished higher education (n = 60, 35.1% for the control, and n = 36, 26.9% for the STI counselling group).

Most risk behaviours were rare, which was quite common in both the control (from 0 to 9.70%) and STI counselling groups (Table 1), but a high proportion of unprotected sex was observed in both groups (80% in the examined and 86% in the control group).

The largest difference was observed in homosexual contact; that is, in the control group, only one participant reported this behaviour (0.6%), while 35 (26.1%) participants in the STI counselling group reported homosexual contact (Table 1).

More participants in the STI counselling group (n = 22, 16.4%) used counselling after testing than those in the control group (n = 1, 0.6%).

Regarding their marital status, participants from the control group were more inclined towards long-term romantic relationships (n = 55, 32.2% married; n = 61, 65.7% in a long-term relationship) compared to participants from the STI counselling group (n = 9, 6.7% married; n = 40, 29.9% in a long-term relationship). More participants from the STI counselling group were single (n = 73, 54.5%) compared to those from the control group (n = 40, 23.4%). Furthermore, more participants from the control group had children (n = 71, 41.5%) compared to those from the STI counselling group (n = 14, 10.5%).

### 3.2. Subjective Risk Assessment of HIV Infection

The subjective risk assessment of HIV infection is presented in Table 2. Generally, the participants in the STI counselling group perceived a higher risk than those in the control group (2.0 (1.0–3.0) vs. 1.0 (1.0–1.0), *p* < 0.001). The majority of participants from the control group reported no risk (n = 155, 93%), while only 35 (26%) from the STI counselling group reported no risk. Only two participants from the STI counselling group reported very high risk, most of whom reported the risk to be low (n = 55, 41.1%) or moderate (n = 30, 22.4%).

### 3.3. Cyberchondria and Health Literacy

The Cyberchondria Severity Scale had an excellent reliability of α = 0.95. Most of the subscales also showed excellent reliability: Compulsion (α = 0.92), Distress (α = 0.95), Excessiveness (α = 0.84), and Reassurance (α = 0.87), but not Mistrust of Medical Professional (α = 0.45). The reliability of the eHealth Literacy Scale was excellent: α = 0.93.

Descriptive statistics and group differences in HL and CH are reported in Table 3.

There was no difference in HL between the groups, and both groups had average HL. However, there were differences in the total CH score and almost all CH factors except for Mistrust. The STI counselling group had average CH, while it was low in the control group. Compulsion was low in both groups but higher in the STI counselling group if analyzing the subscales of the CH scale. Then, Distress was average in the STI counselling group and low in the control group. Excessiveness was average in both groups but higher in the STI counselling group. Reassurance was average in the STI counselling group and low in the control group. Finally, Mistrust was high in both groups, while the other cyberchondria factors were low or average.

There were no sex differences in CH in the STI counselling group (*p* = 0.489) and control group (*p* = 0.137).

In addition, the correlation between HL and CH was significant and positive in both groups: 0.23 (*p* = 0.001) in the examined group and 0.19 (*p* = 0.018) in the control group.

We used a series of logistic regression models investigating CH with predictors: health literacy, belonging to the STI counselling group, subjective risk assessment of HIV infection, demographic variables (age, sex, living alone, having a lower educational level), and risk behaviours (being a homosexual and having sex without a condom) (Table 4). All models were significant except for Mistrust. The chosen predictors best explained the total CH score (R^2^ = 0.26) and Excessiveness (R^2^ = 0.36). Higher health literacy, belonging to the STI counselling group, and carrying out risk behaviours are significantly associated with a higher CH total score. Higher health literacy and belonging to the STI counselling group are associated with almost all of its domains, besides Mistrust. Age, sex, and educational level are not associated with the CH total score and its domains.

## 4. Discussion

Our main hypothesis that participants with a higher risk of STIs have lower HL and higher CH was partially confirmed. Participants in the STI counselling group had a higher CH but also a higher HL than the control group. In addition, although we did not expect that CH would be positively associated with HL, similar results were obtained in the study by Kobryn and Duplaga (2023) [21] and El-Zayat et al. [22] in general populations.

The logistic regression models we made to explain the CH in our participants exhibited a good prediction ability (Table 4), and the CH total score was associated with significant predictors in the STI counselling group—possibility of an STI, higher health literacy, and risk behaviours. Although the perception of risk was not a significant predictor (the participants did not perceive their risk of STIs to be high), they did come to the VCT centre, and their CH was higher than in the control group [23]. The best model was for the Excessiveness factor (unnecessary and repeated internet searches), and health literacy was the strongest predictor. It could be deduced that the higher the level of HL, the stronger the urge for excessive internet browsing for health-related information. This is in accordance with findings by El-Zayat et al. on cyberchondria and its association with smartphone addiction and high eHealth literacy [22].

Higher health literacy and belonging to the STI counselling group are significantly associated with a higher degree of CH and all of its domains, in addition to Reassurance and Mistrust. This finding may be connected to the fact that the survey was performed during the COVID-19 pandemic. Namely, people worldwide were confronted with medical information that was changing rapidly on a daily basis, increasing doubt in the organization and effectiveness of healthcare services and strengthening fear and uncertainty, especially regarding vaccination [23]. This is in accordance with findings by Kobryn and Duplaga (2024) on the cyberchondria score being a negative predictor of COVID-19 vaccine uptake [24]. In addition, the Mistrust factor had a lower reliability, which is also similar to previous results [18] and probably, as a factor, does not belong to the total CH score.

In our research, in the STI counselling group, there were more male than female participants, while the gender distribution in the control group was quite even. The results show that the gender distribution is not random in the control and STI counselling groups. One of the explanations as to why men are more present in the STI counselling group may be the difference in the decision-making process, including the decision to access testing and counselling. It is considered that men use a more logical approach, focused on universal moral standards, and emphasize duty and justice, characterized by individual responsibility towards themselves and others, during the decision-making process [25,26,27]. Another reason could be the higher prevalence of HIV and other STIs in males than in females, and therefore a higher percentage of males in the STI group [3]. Our data concur with those of Fasciana et al. on the sociodemographic characteristics and sexual behavioural factors of patients with STIs in one Italian hospital. Their patients were also predominantly men with a high level of education [28].

Most participants from both groups had at least a high school education, and many had finished higher education. Numerous studies have shown a correlation between levels of formal education and the level of the HL, as well as their impact on health promotion and disease prevention [29,30]. Education, health, and HL are interconnected, and higher levels of education might promote better HL and health knowledge [31,32]. Educated individuals will be able to better understand the relevant information that will help them make better health decisions [29,30,33]. Since there were no significant differences in education level between groups, there was also no significant difference in HL, whereby both groups had average HL.

More participants from the STI counselling group were single, while more participants from the control group were married or were in a long-term relationship and had children. Being single was associated with Compulsion and Distress factors. Sociodemographic and socioeconomic factors, one’s personality (optimism, control over life), and social involvement (social capital) must be included when discussing one’s dimension of health [34]. They all represent potential risks or protective factors for one’s health. Thus, members of the control group with stronger social capital were less inclined to have STIs.

A high proportion of unprotected sex was observed in both groups, and interestingly, there was a low perception of the risk of STIs. The low perception may have been obtained because this study was carried out during the COVID-19 pandemic and other diseases were considered less serious. The participants in the STI counselling group perceived the risk for HIV infection to be higher than the participants from the control group (Table 2), but the perceived risk was still very low. The largest difference in groups was observed in homosexual contact, which was more often reported in the STI counselling group, which was also an expected result. Fasciana et al. also reported poor use of condoms and a high number of partners in their research. MSM had the highest sex-behaviour-related risk for STIs [28]. More participants from the STI counselling group used counselling after testing than participants from the control group. Reliable information and effective communication are enabled through the VCT setting. Namely, various studies recommend that STI teams need to implement counselling topics and recommendations to share with patients, as well as tips on how to approach sexual health education and counselling [28].

In September 2016, the World Health Organization (WHO) Regional Office for Europe created the “Action plan for sexual and reproductive health: towards achieving the 2030 Agenda for Sustainable Development in Europe—leaving no one behind (RC66)”, which aims to improve sexual and reproductive health in Europe. The action plan highlights the importance of the following: enabling informed decisions about one’s sexual and reproductive health and ensuring the respect, protection, and enforcement of human rights; ensuring the availability of the highest possible standards of sexual and reproductive health and well-being; guarantees of universal access to sexual and reproductive health, and the elimination of inequality. The cooperation of international and national partners, relevant ministries, and the non-governmental sector is key to the successful implementation of the Action Plan [35]. All individuals have the right to make decisions governing their bodies and to access services that support that right. It has been proven that access to high-quality, affordable sexual and reproductive health services and information saves lives, improves health and well-being, and promotes gender equality [36]. However, different authors have pointed out for many years that successful health policies must holistically approach both the individual and the community and observe them in their biological and cultural totality. Thus, for example, Dahlgren and Whitehead argued in the early 1990s that the factors that make up one’s lifestyle largely depend on social, environmental, and working conditions, and that one’s health status does not depend solely on individual choice, but is a consequence of the conditions in which one as an individual is born and raised and lives, works, and ages [37].

In seeking, understanding, and evaluating online sexual health information, gender, sexual identity, stigma, structural factors, and social support are important factors in understanding sexual health literacy, especially in young people [38].

It is also considered that, in the healthcare system, there is a so-called “information asymmetry” between users and providers of health services [39]. HL currently extends beyond the ability to simply understand health-related terms and concepts; it encompasses problem-solving abilities, communication skills, and proficiency in using information technologies [40]. The growing significance of the internet’s influence on medical decision-making processes could be observed through the suggestion of the expansion of the biopsychosocial model of health and disease. Namely, along with biological, psychological, and social factors that contribute to health and disease, the model should also contain the digital factor, which may influence all of the aforementioned factors [41]. 

The WHO has coined the term “infodemic” to describe not only the rapid spread of misinformation but also the health risks associated with disinformation [42]. In building resilience against disinformation, user-friendly digital applications and information resources that ensure data privacy and security and deliver genuine benefits to one’s health should be developed [39].

This study has a few limitations. Our sample was convenient, and this study was carried out in the time of the COVID-19 pandemic when people were more preoccupied with COVID-19-related medical problems than with other diseases, and therefore, the perception of STIs was lower. The method itself, the questionnaire, is biased, and the topic is sensitive, so the participants could have given socially desirable answers, and the scales we used do not have a lie scale. In addition, the personalities of the participants and their other diseases as potential predictors of CH remain unknown. However, while the majority of reported research was conducted on healthy participants, this was exactly one of the advantages of our study.

## 5. Conclusions

In our study, people at risk of STIs tend to minimize that risk and have higher CH levels and average HL. If average HL enables individuals to gain access to, understand, and use medical information to promote and maintain good health, but at the same time positively correlates with cyberchondria, then more education about excessive health-related internet research is needed in order to minimize the risk of mental health problems.

## Figures and Tables

**Table 1:** Participants’ characteristics.

Variable	STI Counselling Group (n = 134)	Control Group (n = 171)	*p*
	N (%) or M (SD)	N (%) or M (SD)	
**Gender**			
Female	42 (31.3)	86 (50.3)	0.002
Male	88 (65.7)	85 (49.7)	
Missing	4 (2.9)	0 (0.0)	
**Age**	32.2 (9.8)	36.2 (11.6)	0.002
**Educational level**			
Unfinished elementary school	2 (1.5)	0 (0.0)	
Elementary school	1 (0.8)	4 (2.3)	0.149
High school	87 (64.9)	105 (61.4)	
Higher education	36 (26.9)	60 (35.1)	
Missing	8 (5.9)	2 (1.2)	
Risk behaviour *			
Drug use	6 (4.5)	0 (0.0)	
Prostitution	8 (5.9)	2 (1.2)	
Promiscuity	13 (9.7)	6 (3.5)	
Unprotected sex	106 (79.1)	147 (85.9)	<0.001
Homosexual contact	35 (26.1)	1 (0.6)	
Other	3 (2.2)	3 (1.8)	
Missing	16 (11.9)	10 (5.9)	
Counselling after testing *	22 (16.4)	1 (0.6)	/
**Marital status**			
Married	9 (6.7)	55 (32.2)	
Single	73 (54.5)	40 (23.4)	
Long-term relationship	40 (29.9)	61 (35.7)	<0.001
Divorced	7 (5.2)	11 (6.4)	
Separated	0 (0.0)	1 (0.6)	
Widow(er)	0 (0.0)	1 (0.6)	
Missing	5 (3.7)	2 (1.2)	

Legend: N—number of participants; %—relative frequency of participants. * Frequencies and percentages refer to those that responded “yes” to risk behaviour questions.

**Table 2:** Subjective risk assessment of HIV infection.

Risk Assessment	STI Counselling Group (n = 128) n (%)	Control Group (n = 167) n (%)	*p*
No risk	35 (26.1)	155 (92.8)	
Low risk	55 (41.1)	9 (5.4)	
Moderate risk	30 (22.4)	2 (1.2)	<0.001 *
High risk	6 (4.5)	0 (0.0)	
Very high risk	2 (1.5)	1 (0.6)	
Median (25–75 percentiles)	2.0 (1.0–3.0)	1.0 (1.0–1.0)	<0.001 **

Legend: *—chi square test; **—Mann–Whitney test.

**Table 3:** Descriptive statistics and group differences in health literacy and cyberchondria factors.

	STI Counselling Group		Control Group		Statistics
Variable	n	M (SD)	n	M (SD)	*p*
Health literacy	133	27.88 (6.7)	171	26.49 (7.8)	0.090
Cyberchondria total score	123	89.6 (21.5)	159	72.3 (21.0)	<0.001
Compulsion	128	1.79 (0.7)	165	1.41 (0.5)	<0.001
Distress	129	2.54 (0.9)	163	1.90 (0.9)	<0.001
Excessiveness	128	3.23 (0.8)	165	2.58 (0.9)	<0.001
Reassurance	129	2.95 (0.9)	165	2.27 (0.9)	<0.001
Mistrust	129	4.47 (0.6)	166	4.50 (0.7)	0.700

Legend: M—mean; SD—standard deviation; *p*—two-tailed probability for Welch’s test. CH subscales: results were considered low at 1–2.33, average at 2.34–3.67, and high at 3.68–5.00.

**Table 4:** Logistic regression models investigating independent contributions of health literacy, being in the STI counselling group, subjective risk assessment, and other demographic and risk variables to Total Cyberchondria, Compulsion, Distress, Excessiveness, Reassurance, and Mistrust (n = 283).

Variable	CH	Compulsion	Distress	Excessiveness	Reassurance	Mistrust
	OR (95% CI) *p*					
Health literacy	1.08 (1.04–1.14) <0.001	1.03 (0.99–1.07) 0.117	1.06 (1.03–1.10) 0.002	1.15 (1.10–1.21) <0.001	1.05 (1.01–1.09) 0.013	1.00 (0.97–1.04) 0.959
STI counselling group	3.25 (1.54–6.87) 0.002	2.98 (1.47–6.06) 0.002	2.51 (1.24–5.09) 0.010	4.35 (1.97–9.60) <0.001	1.89 (0.96–3.73) 0.065	1.59 (0.81–3.14) 0.177
Subjective risk assessment of HIV infection	0.72 (0.45–1.14) 0.160	0.67 (0.43–1.03) 0.073	0.87 (0.57–1.33) 0.537	0.73 (0.46–1.18) 0.202	1.25 (0.83–1.88) 0.285	0.77 (0.51–1.14) 0.194
Age	0.98 (0.95–1.00) 0.186	0.99 (0.97–1.02) 0.947	0.99 (0.96–1.01) 0.507	0.98 (0.96–1.02) 0.482	0.99 (0.96–1.01) 0.288	0.99 (0.97–1.01) 0.415
Sex	1.18 (0.65–2.14) 0.584	1.18 (0.67–2.08) 0.561	1.37 (0.77–2.45) 0.273	0.94 (0.51–1.74) 0.839	1.71 (0.97–3.00) 0.064	0.96 (0.56–1.65) 0.895
Living alone	1.54 (0.80–2.94) 0.187	2.41 (1.31–4.45) 0.004	2.00 (1.07–3.72) 0.029	1.59 (0.82–3.09) 0.167	1.23 (0.67–2.26) 0.501	0.56 (0.31–1.01) 0.055
Low educational level	0.62 (0.03–13.8) 0.760	0.62 (0.03–13.8) 0.760	0.81 (0.05–13.4) 0.888	0.95 (0.03–25.70) 0.978	0.54 (0.03–11.09) 0.691	2.58 (0.22–30.5) 0.450
Being a homosexual	2.92 (1.01–8.56) 0.049	2.08 (0.77–5.60) 0.147	1.80 (0.67–4.80) 0.238	1.98 (0.70–5.63) 0.198	2.26 (0.87–5.96) 0.098	0.54 (0.21–1.36) 0.193
Having sex without a condom	0.34 (0.11–0.99) 0.049	0.81 (0.30–2.14) 0.067	0.37 (0.14–1.01 0.053	0.45 (0.16–1.24) 0.123	0.35 (0.13–0.92) 0.033	0.54 (0.21–1.36) 0.203
Nagelkerke R^2^ *p*	0.26 <0.001	0.18 <0.001	0.20 <0.001	0.36 <0.001	0.17 <0.001	0.053 0.324

Legend. β: CH—recoded cyberchondria total score (0 < 78; 1 ≥ 78), recoded Compulsion (0 < 1.33; 1 ≥ 1.33), recoded Distress (0 < 2.12; 1 ≥ 2.12), recoded Excessiveness (0 < 3.00.; 1 ≥ 3.00), recoded Reassurance (0 < 2.50; 1 ≥ 2.50), and recoded Mistrust (0 < 4.5; 1 ≥ 4.5); OR (95% CI)—odds ratio with 95% confidence interval; low educational level—did not finish high school.

## Data Availability

The data presented in this study are available on reasonable request from the corresponding author.
